# NLRP3 Q703K and TNFRSF1A R92Q mutations in a patient with auto inflammatory disease

**DOI:** 10.1186/1546-0096-13-S1-P47

**Published:** 2015-09-28

**Authors:** I von Mühlenen, C Gabay, A Finckh, J Kuemmerle-Deschner

**Affiliations:** 1 Rheuma-Basel, Basel, Switzerland; 2 HUGE, Rheumatology, Geneve, Switzerland; 3 Universitätsklinikum Tübingen, Pädiatrische Rheumatologie, Tübingen, Germany

## Background

Cryopyrin-associated periodic syndrome (CAPS) and tumor necrosis factor receptor-associated periodic syndrome (TRAPS) are rare autoinflammatory disorders caused by NLRP3 and TNFRSF1A mutations. The Q703K (CAPS) and the R92Q (TRAPS) variants however, are considered to be low-penetrance variants with little or no clinical significance [1,2].

## Objective

To describe the clinical presentation and disease course in a young man with the Q703K and the R92Q mutations.

## Methods

Thorough clinical and laboratory examination and genetic testing were carried out in a patient presenting with autoinflammatory symptoms.

## Results

At the age of 2 the patient was hospitalized for recurrent fever (39 - 41°C) during several months. Recurrent infections were suspected, although presence of bacteria or virus could not be confirmed. Clinical examination revealed multiple lymph nodes and enlarged tonsils. At the age of 4, the boy was hospitalized because of chronic abdominal pain. At the age of 5, neurological development became abnormal and psychomotor development deficiency was diagnosed. By the age of 8 symptoms included ataxia, abdominal pain, headache, fatigue, arthralgia and myalgia. Neurological symptoms were rapidly progressive. Within few months the patient was unable to walk. In addition, pyoderma grangrenosum-like skin lesions appeared. After several months, slow improvement was noticed. He was 15 years old when he presented with an episode of periorbital edema and fever. At the age of 18 recurrent fever, headache and abdominal pain persisted. The laboratory tests showed elevated CRP and IgD, but no signs of infection. Periodic fever syndrome was suspected and genetic analysis confirmed the presence of a Q703K and R92Q Mutation.

## Conclusion

The course of disease in this patient suggests, that the presence of a Q703K and R92Q mutation, although each known as low-penetration variants, may be clinically significant and consistent with an autoinflammatory syndrome.

## Consent to publish

Written informed consent for publication of their clinical details was obtained from the patient/parent/guardian/relative of the patient.
